# The molecular architecture of *Drosophila melanogaster* defense against *Beauveria bassiana* explored through evolve and resequence and quantitative trait locus mapping

**DOI:** 10.1093/g3journal/jkab324

**Published:** 2021-09-17

**Authors:** Parvin Shahrestani, Elizabeth King, Reza Ramezan, Mark Phillips, Melissa Riddle, Marisa Thornburg, Zachary Greenspan, Yonathan Estrella, Kelly Garcia, Pratik Chowdhury, Glen Malarat, Ming Zhu, Susan M Rottshaefer, Stephen Wraight, Michael Griggs, John Vandenberg, Anthony D Long, Andrew G Clark, Brian P Lazzaro

**Affiliations:** 1 Department of Biological Science, California State University Fullerton, Fullerton, CA 92831, USA; 2 Division of Biological Sciences, University of Missouri, Columbia, MO 65211, USA; 3 Department of Statistics and Actuarial Science, University of Waterloo, Waterloo, ON N2L 3G1, Canada; 4 Department of Integrative Biology, Oregon State University, Corvallis, OR 97331, USA; 5 Department of Ecology and Evolutionary Biology, University of California Irvine, Irvine, CA 92692, USA; 6 Department of Entomology, Cornell University, Ithaca, NY 14853, USA; 7 USDA ARS Emerging Pets and Pathogens Research Unit, Robert W. Holley Center for Agriculture & Health, Ithaca, NY 14853, USA; 8 Department of Molecular Biology and Genetics, Cornell University, Ithaca, NY 14853, USA

**Keywords:** *Drosophila melanogaster*, *Beauveria bassiana*, immunity, DSPR, QTL mapping, E&R, experimental evolution

## Abstract

Little is known about the genetic architecture of antifungal immunity in natural populations. Using two population genetic approaches, quantitative trait locus (QTL) mapping and evolve and resequence (E&R), we explored *D. melanogaster* immune defense against infection with the fungus *Beauveria bassiana*. The immune defense was highly variable both in the recombinant inbred lines from the Drosophila Synthetic Population Resource used for our QTL mapping and in the synthetic outbred populations used in our E&R study. Survivorship of infection improved dramatically over just 10 generations in the E&R study, and continued to increase for an additional nine generations, revealing a trade-off with uninfected longevity. Populations selected for increased defense against *B. bassiana* evolved cross resistance to a second, distinct *B. bassiana* strain but not to bacterial pathogens. The QTL mapping study revealed that sexual dimorphism in defense depends on host genotype, and the E&R study indicated that sexual dimorphism also depends on the specific pathogen to which the host is exposed. Both the QTL mapping and E&R experiments generated lists of potentially causal candidate genes, although these lists were nonoverlapping.

## Introduction

Studies of insect immune defense have focused predominantly on immune mechanisms against bacteria and viruses, while defense against entomopathogenic fungi remains poorly understood. Understanding the molecular architecture of insect susceptibility to fungal entomopathogens has potential to guide biological control efforts. *Beauveria bassiana* is an entomopathogenic fungus that has been used to control crop pests that threaten food security for a growing international human population (Ugine *et al.* 2005; Li *et al.* 2010). Fungal biocontrol also has major prospective public health impact through suppression of disease vector insects. For example, *B. bassiana* can be deployed for management of bed bugs (Barbarin *et al.* 2012) and has potential to limit mosquitos in the genera *Aedes and Anopheles* (García-Munguía *et al.* 2011; Valero-Jiménez *et al.* 2017). Susceptibility to *B. bassiana* is genetically variable within populations of insects (Tinsley *et al.* 2006). This variability can provide the substrate for natural selection to increase resistance, which may thwart control initiatives. Given the broad host range of *B. bassiana*, this pathogen is unlikely to be in any strict coevolutionary arms races with its varied hosts. Thus, we can effectively use the fruit fly, *Drosophila melanogaster*, which shares many homologous immune defense genes and pathways with other insects as a model for defense against this generalist fungus.

Studies with *D. melanogaster*, have already identified several genes and pathways involved in insect immune defense against fungal pathogens (Buchon *et al.* 2014), but much of the inter-individual variation in immune defense against fungal infection still remains unexplained. Here, we used two approaches for identifying causative loci underlying variation in *D. melanogaster* defense against *B. bassiana*: QTL mapping, and evolve and resequence (E&R). For our QTL mapping study, we used a large multiparental advanced generation reference panel called the Drosophila Synthetic Population Resource (DSPR) (http://FlyRILs.org; last accessed 9/18/21). The DSPR panel allows the estimation of effects of alleles that are at low frequency in natural populations, as long as these variants are present in the initial founder lines (Macdonald and Long 2007). Moreover, the DSPR has high power and high mapping resolution (King *et al.* 2012a). The DSPR has already been used to examine the genetic bases for various traits (reviewed by Long *et al.* 2014), including pathogen susceptibility (Duxbury *et al.* 2019), caffeine and boric acid resistance (Najarro *et al.*^2015^, ^2017^), and methotrexate toxicity (Kislukhin *et al.* 2013). Using post-infection survival as a measure of immunity in the DSPR, we identified several genomic positions correlated with immunity. Males and females survived infection differently, with the direction and magnitude of sexual dimorphism being dependent on fly genotype.

In natural populations, alleles with large effects on *D. melanogaster* immune defense, of the type that can be identified via QTL mapping, may be selected against due to pleiotropy or trade-offs with other fitness characters. It is unclear whether there is substantial rapidly selectable variation in defense against *B. bassiana* in natural *D. melanogaster* populations. We used laboratory selection to show that populations of *D. melanogaster* can evolve rapidly to become more resistant to *B. bassiana* and identified the alleles that changed in frequency over the course of this evolution. Evolved improvements in immune defense came at a cost to uninfected longevity, demonstrating the existence of an evolutionary trade-off. Cross-resistance tests showed that selection for defense against *B. bassiana* did not affect resistance against bacterial pathogens.

## Materials and methods

### Quantitative trait locus mapping

#### The Drosophila Synthetic Population Resource (DSPR)

We phenotyped 296 RILs from the A1 population of the DSPR (King *et al.*2012a, 2012b). For at least 8 generations prior to being phenotyped for immune defense, the RILs were moved to Cornell Biotech Glucose medium (per liter of deionized water: 82 g glucose, 82 b Brewer’s yeast, 10 g agar, 10 mL acid mix composed of 4.15% phosphoric acid by volume).

#### Preparation of RILs for phenotyping

Prior to each assay, 100 male and 100 female flies per RIL were anesthetized with carbon dioxide (CO_2_) and placed in a 14 oz bottle with a small Petri dish of medium supplemented with yeast paste (approximately 1 teaspoon yeast mixed in 1 mL DI water). After 24 h eggs were separated into 8-dram vials at densities of 60–80 eggs/vial and given ∼13 days to emerge into adults. Adults were then transferred to fresh vials daily until inoculation.

Sexes were mixed in the vials. For each RIL this process was repeated three separate times over the span of a year, with the exception of 13 RILs that were tested only twice. As such, there were 2–3 biological replicates when measuring each RIL’s immune defense. We randomized the timing of the replicate tests: a random “group” of ∼50 RILs underwent egg collection on the same day. Then on day 16 from egg, a random “set” from each group, comprising ∼25 RILs were inoculated. The next day, on day 17 from egg, a second set of ∼25 RILs from the same group were inoculated. The three replicates tested for each RIL were tested in different *groups* (with the exception of 36 RILS for which some replicates were tested in the same group). Thus, RILs in the same “*group*” were reared together, and RILs in the same *set* (nested within group) were sprayed together. The division into groups and sets was necessary for handling because 878 inoculation assays were performed in total.

#### Inoculation of flies with B. bassiana

Flies were inoculated with *B. bassiana* ARSEF 12460 Shahrestani & Vandenberg (Shahrestani *et al.* 2018). Flies were briefly anesthetized with CO_2_ and measured in a microcentrifuge tube to 0.5 mL, which corresponds to approximately 50 flies/sex. These 100 flies were then spread out on a small Petri plate lid placed on ice to sustain anesthetization. Flies were sprayed with 5 mL of a fungal suspension (0.034 g spores/25 mL of 0.03% Silwet) using a Spray Tower (Vandenberg 1996), which introduced approximately 100 spores/mm^2^ of *B. bassiana* to the fly cuticle. Inoculated flies were placed into cages and kept at 25°C and 100% humidity for 24 h to allow the fungus to germinate. Each cage contained 100 flies (∼50 male and 50 female) that were sprayed together. Afterward, the cages were maintained at 25°C at 60–70% humidity with the usual 12/12 h-light/dark cycle. Mortality was counted daily and recorded for 10 days distinguishing the number of males and females that were dead or lost due to handling. After 10 days, the surviving flies were terminated and counted to determine the exact number of flies that were in each cage. Survival 10-days post inoculation was used as the focal phenotype (Supplementary File S2 includes raw phenotype data for the RILs).

#### Fungal viability check and spore count

Dried spores were stored at minus 20°C until checked for viability or dosing of insects. On the same day as each spray, we confirmed spore viability. A 2 mL suspension of a 1:1000 dilution of 0.34 g of lab grown *B. bassiana* in 25 mL of 0.03% Silwet was sprayed through the spray tower onto a 60 × 15 mm water agar Petri dish which was incubated at 25°C for 24 h. Following incubation one hundred spores within a central swath were inspected under a light microscope for presence or absence of a growing germ tube. Spores with a germ tube greater than or equal to the length of the spore were tabulated as living while others were considered nonviable. The ratio of living vs nonliving spores was used to determine % viability. In our study percentage viability was always >90%. To estimate deposition of spores on the arena a plastic microscope cover slip (22 × 22 mm) was placed adjacent to each group of insects sprayed with a dose. After the cover slips were dried they were placed in a 50 mL centrifuge tube with approximately 15 small glass beads and 5 mL of 0.03% Silwet in autoclaved DI water. A vortex shaker was used to dislodge spores from the coverslip into suspension (Ugine *et al.* 2005). Using a pipette, a drop of spore suspension was placed onto two hemocytometers. Spores were counted to estimate number of spores per mL deriving the number of spores/mm^2^ deposited on the fly spray arena. In our study, spores/mm^2^ were always predictable from our initial suspension and we found no reason to exclude any data due to spore viability or dosage concerns.

#### Data analysis

All data analysis was carried out using the R statistical programming language (R Core Team 2017). We tested for the overall effects of RIL, sex, and a RIL × sex interaction, as well as the effects of vial, group, and set on survival by fitting a set of generalized linear mixed models using the lmer function in the lme4 package (Bates *et al.* 2015) with a binomial distribution. The model is as follows:

Survival~sex+ (1|patRIL) + (1|RIL:vial) + (1|RIL:sex) + (1|group) + (1|set:group)

The fixed effect of sex was highly significant (*z* = −8.0, *P* < 0.001). For the random effects, we performed likelihood ratio tests to obtain *P*-values comparing the full model shown above to a reduced model not including each random effect using the ANOVA function. The effects of vial (nested in RIL), RIL, and the RIL × sex interaction were all significant as well (vial: $\chi_{1}^{2}$ = 478.5, *P* < 0.001; RIL: $\chi_{1}^{2}$ = 313.9, *P* < 0.001; RIL × sex: $\chi_{1}^{2}$ = 1701.6, *P* < 0.001). Both group and set (nested in group) were not significant (group: $\chi_{1}^{2}$ = 3.28, *P* = 0.07; set: $\chi_{1}^{2}$ = 1.55, *P* = 0.21) and dropping these did not appreciably affect the effect estimate of the fixed effect (sex). Given this, and the fact that for the vast majority of RILs, replicates were distributed among three groups, we did not include group or set in any future analyses. To estimate broad-sense heritability in the set of RILs, we fit the following model separately for males and females using the lmer function:

$$\text{Survival \textasciitilde{} 1 + }\left(\text{1|patRIL}\right)\text{ + }\left(\text{1|patRIL:vial}\right)\text{.}$$

We calculated variance components using the VarCorr function. Because survival is a 0/1 trait, we used a threshold model to calculate the broad-sense heritability (see Roff 1997) in the RILs.

#### QTL mapping

The focal phenotype for each RIL for QTL mapping is the average proportion surviving the fungal infection 10 days post-inoculation. The distributions of phenotypes were slightly skewed (Supplementary Figure S1). Thus, to improve normality, we used an arcsine square root transformation, commonly used to transform proportion data (Supplementary Figure S1). The methodology for mapping QTL in the DSPR, and in multiparent populations more generally, has been described extensively previously (Broman and Sen 2009; King *et al.*2012a, 2012b). Briefly, a hidden Markov model assigns the underlying founder ancestry to each segment of each RIL with an associated probability (King *et al.* 2012b). At each of ∼10,000 positions across the genome, we regressed the focal line phenotype on the eight founder probabilities, analyzing males and females separately. We also mapped the difference between male and female survivorship to examine dimorphism in susceptibility. We obtained qualitatively similar results to a global analysis including both males and females and a haplotype by set interaction with RIL as a random grouping variable. We chose to present the results from the analysis of the sexes separately for ease of interpretation as well as the increased simplicity of the model, which makes using a permutation test more feasible. We used a permutation test (Churchill and Doerge 1994) to identify the genome-wide significance threshold associated with a 5% family-wise error rate and to estimate the false discovery rate (FDR) (Benjamini and Hochberg 1995). For each permutation, we calculated the average number of false QTL across all phenotypes at different significant thresholds. We first identified all peak positions for a given genome scan, then we removed any peaks that were within 2 centiMorgans of a higher peak. We then calculated the threshold that corresponded to the FDR. Here the FDR is the expected number of false positives/the expected number of total positives at a threshold of 5% and 50%. To calculate a confidence interval for each QTL peak, we used a 2-LOD drop (King *et al.* 2012a). We used FlyBase (www.flybase.org) to convert the 5.x coordinates given by the QTL analysis to the updated 6.x coordinates. Then we used FlyBase to identify the genes located in these regions of interest.

### Experimental evolution and resequencing

#### Selection protocol

The base population was obtained from co-author AGC and was originally created by combining 96 isofemale lines from five geographic areas in order to maximize genetic diversity (Greenberg *et al.* 2010). The lines were from Beijing (Begun and Aquadro 1995), Netherlands (Bochdanovits and Jong 2003), Ithaca NY, Tasmania, and Zimbabwe (Begun and Aquadro 1993). The outbred population was maintained on discrete 14-day generations in a 12:12 light/dark incubator, with developmental phase in bottles and egg-laying phase in cages for more than 50 generations prior to the start of our study. After increasing the population size of the base population slowly over six generations, we divided it into eight groups and randomly assigned these groups to four control (C_1__–__4_) and four selected populations (S_1__–__4_). For each of the eight populations, ∼10,000 eggs were collected per generation. In the S_1__–__4_ populations, these ∼10,000 flies (estimated by volume: 2000 adult flies was approximately 5 ml of volume) were inoculated with *B. bassiana* (see protocol below) and then divided into five cages at densities of 2000 flies per cage. For each of the four C populations, ∼10,000 eggs were collected and after eclosion, ∼2000 adults were randomly chosen, control sprayed (see protocol below) and placed into a single cage. Dead flies were removed from the cages daily, preventing secondary infection from cadavers in the fungal inoculated groups. Flies were fed daily with Petri plates filled with medium. When 80% of the flies in an S population died, surviving flies across all five cages for that population were combined to form a single cage with approximately 2000 flies and given yeast supplement in addition to the diet to promote oviposition. Over the following 1–3 days, 100 vials of eggs at densities of ∼60–80 eggs/vial were collected for each population to yield over 10,000 eggs per population. This protocol was repeated for 19 generations. We directly tested for, and found no evidence for transgenerational carry over of fungal spores (data not shown). Each *C_i_* population was kept on the same timing as its corresponding S_i_ population.

#### Beauveria bassiana inoculations and verification of dose and viability

For selection on immune defense, we inoculated flies in the same way as in the QTL mapping study described above, except every generation flies were inoculated with 7.5 ml of 0.34 g of *B. bassiana* spores suspended in 25 ml of 0.03% Silwet in DI water. This is a higher dose (∼1000 spores/mm^2^) than in the QTL study for two reasons: the outbred population was more robust than most of the RILs used in the QTL mapping study, and we wanted ∼80% of inoculated flies to die within two weeks. For the dose response assays we diluted this suspension to 1:10, 1:100, and 1:1000. Viability and dose of *B. bassiana* were checked using the protocols described above. The selection dose (undiluted) was ∼10^4^ spores per mm^2^, and the fungus was viable (>90%) in all sprays. In each infection assay, some of the flies that died were plated on SDAY media and monitored for fungal sporulation on their cuticle, confirming that flies had been infected. Control groups underwent the same treatment as the inoculated groups, but were sprayed with just 0.03% Silwet suspension (no fungus). Cadavers from the control groups did not show sporulation on their cuticle.

#### Infection resistance assays

At generations 10 and 19, we compared the C_1-4_ and S_1-4_ populations for divergence in resistance to four pathogens: *B. bassiana* ARSEF 12460, *B. bassiana* GHA, *Providencia rettgeri*, and *Enterococcus faecalis* (Supplementary Files S2−S4 include raw data for these phenotypes). For the two fungal pathogens and for their matched uninfected control, the sample sizes were ∼100 flies per sex per population, tested in two replicate cages. Flies were handled in the same manner as in the selection protocol, except they were kept in smaller cages to control for density effects, and dead flies were removed and sexed daily until all flies died. For the two bacterial pathogens the sample sizes were ∼100 flies per sex per population, and ∼40 flies per sex per population for the wounding control groups. These flies were anesthetized in groups of 15 or fewer on CO_2_ and pricked in the thorax with a needle dipped in dilute bacterial culture, or with a sterile needle as a wounding control, and then maintained in groups of 10 in vials (Khalil *et al.* 2015). Bacterial cultures were grown overnight in Luria broth at 37°C from a single bacterial colony, and then diluted with sterile LB to O.D._600_ = 1.0 (for *P. rettgeri*) or O.D._600_ = 0.5 (for *E. faecalis*).

#### Statistical analyses for phenotypes

The Kaplan-Meier estimate of the survival function (Kaplan and Meier 1958) and tests of significance using Cox Proportional Hazard model (Cox and Lewis 1972) were performed using the package Survival in R. Survival after infection with *B. bassiana* ARSEF 12460 was analyzed with the following model:

coxph=P + G+D + P*G + P*D + G*D + P*G*D

where * shows interaction, G represents generation (10 and 19), P represents populations (Control and Selected), and D represents the infection status (uninfected and infected with 10^4^ spores/mm^2^). The variables of population replicate, treatment replicate, and sex, did not significantly affect hazard ratios in preliminary analyses and were omitted from the final model. To study the effect of dose when infected with *B. bassiana* ARSEF 12460, we looked at the LT50, the median lethal time in days, and investigated the effect of population (S *vs* C), sex, generation (10 *vs* 19), and dose on LT50 with ANOVA.

Survival after infection with *B. bassiana* GHA was analyzed with the following model:

(2) coxph=S + P+G + S*P + S*G + P*G+S*P*G

where the notation is the same as for model (1), with the addition of S, which represents sex (female and male). Keeping the three-way interaction term, which was borderline significant, improved the validity of the proportional hazard and hence the fit of the model by producing a more horizontal shape in the plot of the Schoenfeld residuals, which is one of the model diagnostics. Since the interactions of sex with other factors were significant, though the marginal effect of sex was nonsignificant, we kept this term in the model to follow a hierarchical modeling approach. Unlike with ARSEF 12460 and GHA, no difference between S and C populations was observed for resistance to the two bacterial infections (*E. faecalis and P. rettgeri*). The hazard ratios in all sub-populations were 1. To test the significance in the difference between two survival functions, we use the log-rank test whose null hypothesis is that the two survival curves are the same. The *P*-values of log-rank tests to compare survival functions in each sub-group were very high, confirming that there is no evidence of difference between the survival functions, *i.e.*, the Hazard Ratio (HR) = 1 across the board. Therefore, no further analysis was conducted on these data.

#### DNA extraction and sequencing

After zero and 19 generations of selection, uninfected samples of adult female flies were frozen in a −80 freezer. Genomic DNA was extracted from 100 female flies collected from each of nine groups: C_1__–__4_ and S_1__–__4_ populations and one from the founding population. These pools were prepared as standard 200–300 bp fragment libraries for Illumina sequencing and sequenced on Illumina Hi-Seq platforms within the Cornell sequencing core facility.

#### Mapping of reads

Reads were mapped against the *D. melanogaster* reference genome (version 6.14) using BWA (version 0.7.8) (Li and Durbin 2009) using bwa mem with default settings. We filtered and sorted the resulting SAM files for reads mapped in proper pairs with a minimum mapping quality of 20 and converted them to the BAM using the view and sort commands in SAMtools (Li *et al.* 2009). The rmdup command in SAMtools was then used to remove potential PCR duplicates. Average coverage was above 30X or greater for all populations except C3, which was 25X (Supplementary Table S1). Next, bam files for all 9 populations were combined into a single mpileup file once again using SAMtools. The mpileup file was in turn converted to a “synchronized” file using the PoPoolation2 software package (Kofler *et al.* 2011). This file contains allele counts for all bases in the reference genome for each population in a succinct tab delimited file. Lastly, RepeatMasker 4.0.3 (http://www.repeatmasker.org; last accessed 9/18/21) was used to create a gff file containing simple sequence repeats found in the *D. melanogaster* genome version 6.14. PoPoolation2 was then used to make these regions within the sync file.

#### Patterns of SNP variation

A SNP table was created using the sync file described above (Supplementary File S5). We only considered sites where coverage was between 15X and 200X, and for a site to be considered polymorphic we required a minimum minor allele frequency of 2%. We defined a minimum minor allele frequency of 2% across all 9 populations by first combining reads for all populations, then filtering out sites where the MAF was less than 2%. All sites failing to meet these criteria were discarded. Prior to performing any of the analyses described below, we sought to identify cases where a given nucleotide was fixed across all of the S populations but not the ancestral P populations and C populations, which may be expected for strong selection on standing variants. To assess broad patterns of SNP variation in P, C, and S populations, heterozygosity was calculated and plotted over 100 kb nonoverlapping windows directly from the major and minor counts in our SNP table. A *t*-test was also performed to compare mean heterozygosity between the C and S populations. To assess how closely replicate populations resembled one another within the C and S groups, *F_ST_* estimates were also obtained using the formula: *F*_ST_ = (*H*_T_ − *H*_S_)/*H*_T_ where *H_T_* is heterozygosity based on total population allele frequencies, and *H_S_* is the average subpopulation heterozygosity in each of the replicate populations (Hedrick 2011). *F*_ST_ estimates were made at every polymorphic site in the data set for a given set of replicate populations.

#### SNP differentiation

We used two different methods to characterize SNP differentiation between the C and S populations. First, we used the Cochran-Mantel-Haenzsel (CMH) test as implemented in the PoPoolation2 software package to compare SNP frequencies at every polymorphic site in our SNP table between the two groups of populations. CMH tests between our two groups of populations were performed at each of these polymorphic sites. Note that in our experimental design the C and S populations are paired; each generation whenever ∼80% of flies in S_1_ died, eggs were collected from both C_1_ and S_1_, such that C_1_ is the control population to S_1_. Moreover, all populations share the common ancestor. To establish a significance threshold for these tests, we first performed simulations to generate a distribution of P-values associated with a null expectation of genetic drift rather than selection (see “Simulations” section below). Briefly, we generated sets of 8 populations based on allele frequencies in the ancestral P populations, then simulated 19 generations of genetic drift. Within each set of 8 populations, half were randomly assigned as “control” and the remainder were randomly assigned as “selected.” CMH tests were then performed at each polymorphic site between the two groups. This was done 100 times and all the resulting *P*-values were recorded. The quantile function in R was then applied to these *P*-values to define a significance threshold that corresponds to a genome-wide false-positive rate, per site, of 5%. This ultimately resulted in a significance threshold of approximately 3.94 × 10^−18^.

Along with the CMH test, we also compared SNP frequencies between the C and S populations using the quasi-binomial GLM approach suggested by Wiberg *et al.* (2017). Based on their findings, this approach is reported to have lower false-positive and higher true positive rates than the CMH tests. However, it should be noted that our simulation-based approach to correcting for multiple comparisons when using the CMH test resulted in a more stringent significance threshold than what was featured in their work. The quasi-binomial GLM test was implemented using scripts provided by Wiberg *et al.* (2017). As suggested by the authors, allele counts were scaled to the effective sample size (*n_eff_*) as described in Kolaczkowski *et al.* (2011) and Feder *et al.* (2012). As counts of zero can lead to problems when implementing this approach (see Wiberg *et al.* 2017), a count of 1 was added to each allele whenever a zero was encountered. In terms of correcting for multiple comparisons, one of the reported benefits of quasi-binomial GLMs is that they produce the expected uniform distribution of p-values under the null hypothesis which allows for standard methods of correcting for multiple comparisons (Wiberg *et al.* 2017). To that end, instead of the simulation approach used for our CMH tests, we opted to use a Bonferroni correction, and the *q*-value approach (Storey and Tibshirani 2003; Storey *et al.* 2017). We chose these two methods as Wiberg *et al.* (2017) found them to be the most and least conservative approaches, respectively.

#### Gene search

We used FlyBase to search for genes within 25 kb of each of the most significantly differentiated SNPs. We separately used the Gowinda software package (Kofler and Schlötterer 2012) to identify enriched GO terms based on our candidate sites. This analysis was not impacted by the number of SNPs within a given gene. Our list of candidate sites consisted of the 45 significantly differentiated SNPs identified between the C and S populations based on our CMH comparison. The background list contained our complete SNP list based on our previously described SNP calling parameters. A gene annotation file for the *D. melanogaster* reference genome (6.14) was obtained from FlyBase, and a gene set file for relevant GO terms was obtained from FuncAssociate3 (Berriz *et al.* 2009). With these inputs, Gowinda was set to run for 10^6^ simulations with the gene-definition and mode parameters set to “gene.” This analysis identified 132 GO terms with *P*-values < 0.05. This list was then filtered so all GO categories containing less than 2 reference genes were discarded, and the resulting list was run through GO-Module to correct for hierarchical clustering (Yang *et al.* 2011).

#### Simulations

To perform our genetic drift simulations, we used MimicrEE (https://sourceforge.net/projects/mimicree; last accessed 9/18/21), a forward simulation specifically designed to mimic experimental evolution. MimicrEE simulates populations of diploid individuals where genomes are provided as haplotypes with two haplotypes constituting a diploid genome. There are no changes in the demography once the initial population file is submitted and a list of selected loci may be provided. The simulated populations have nonoverlapping generations and all individuals are hermaphrodites (though selfing is excluded). At each generation, matings are performed, where mating success (number of offspring) scales linearly with fitness, until the total number of offspring in the population equals the targeted population size (fecundity selection). Each parent contributes a single gamete to the offspring. As we were only interested in simulating genetic drift, we did not specify any fitness differences between different genotypes. Crossing-over events are introduced according to a user-specified recombination rate. The recombination rates were specified for 100 kb windows and were obtained from the *D. melanogaster* recombination rate calculator v2.2 (Fiston-Lavier *et al.* 2010). As recombination does not occur in male *D. melanogaster*, the empirically estimated female recombination rate was divided by two for the simulations.

The starting populations used in our simulations were generated based on SNP frequencies in the ancestral P population across the 268,272 polymorphic sites along chromosome 3R. Each starting population consisted of 600 individuals. To create each individual’s genotype, two random numbers in the range (0.0, 1.0) were generated at each polymorphic site. These numbers were then compared to the ancestral data’s major allele frequency at the position. If the random number was less than the major allele frequency, the major allele was added. Otherwise, the minor allele was added. In this manner, we generated 100 sets of populations each consisting of 8 populations derived from the SNP frequencies in the P populations. All sets of populations were then subjected to 19 generations of drift. Within each set, the populations were then randomly split into two groups of 4 and the CMH tests were performed at each polymorphic site between the two groups. All *P*-values were recorded and, the quantile function in R was used to define a significance threshold that corresponds to a genome-wide false-positive rate, per site, of 5%.

We estimated effective population size (*N_e_*) in our experimental populations using the Nest package in R, which was specifically designed to estimate *N_e_* from temporal allele frequency changes in experimental evolution (Jónás *et al.* 2016). This analysis suggested *N_e_* to be ∼600, so we simulated populations of 600 individuals. As genotype inputs, we used SNP frequencies in P populations for the first time point and frequencies in the C populations for the second. We used the “P.planI” method for estimating *Ne*, which was specifically to account for the two stage sampling process associated with pool-seq data (individuals being sampled from a population, and reads being sampled from DNA pool). *Ne* estimates varied depending on which of the C populations was used, but the average was 592 or ∼600 (N_e_ was 466 for C_1_, 616 for C_2_, 590 for C_3_, and 696 for C_4_).

## Results

### Quantitative trait locus mapping

**Figure 1:**
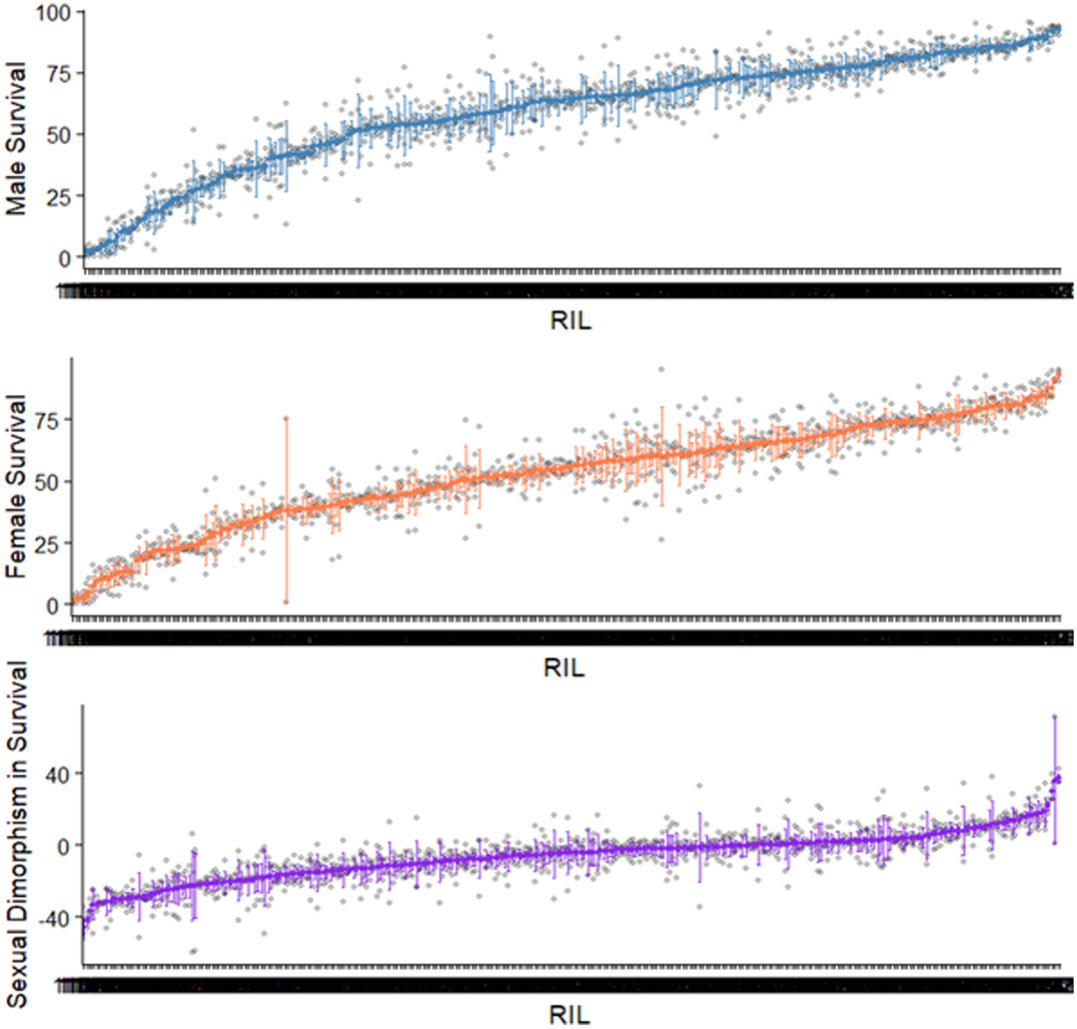
Post-infection survival in the DSPR RILs. Percent of flies surviving in each RIL 10 days post inoculation with *B. bassiana* ARSEF 12460 is shown for males (A) and females (B). Male survival subtracted from female survival gave a measure of sexual dimorphism in survival (C). In some RILs males survived better than females, in others females survived better than males, and in some there was no sexual dimorphism. Bars show standard error of the mean as each RIL was tested multiple times.

We measured survival 10 days after inoculation with *B. bassiana* in 297 RILs from the DSPR. Survival ranged from 0% to 92.25% (Figure 1). For males, mean survival was 58.97% and median survival was 63.77%, and for females the mean and median survivals were 52.45% and 53.93%, respectively. The replicates tested for each RIL were similar to each other (see standard errors of the means in Figure 1); the RIL with the large differences among replicates was RIL11039, which may have been more affected by slight environmental differences among replicates. Broad-sense heritability of survival in the RILs was 0.53 for males and 0.48 for females. Note that in inbred lines, the heritability will be inflated relative to an outbred population. These estimates are consistent with the finding of a highly significant effect of RIL in the overall model.

Most RILs had a sexually dimorphic response to infection, with males and females exhibiting different probabilities of surviving 10 days after infection (Figure 1). Moreover, the magnitude and even the direction of the sexual dimorphism varied across RILs (Figure 1). In 68.35% of the RILs, on average more females than males were dead 10 days after infection, and in the remaining RILs the direction of dimorphism was reversed (Figure 1). Within RILs, the direction of dimorphism remained consistent across replicates. Across RILs, immune defense was positively correlated (*r*^2^ = 0.59; *P* < 2.2 × 10^−16^) between males and females indicating sex-independent genetic differences among the lines (Supplementary Figure S2).

**Figure 2:**
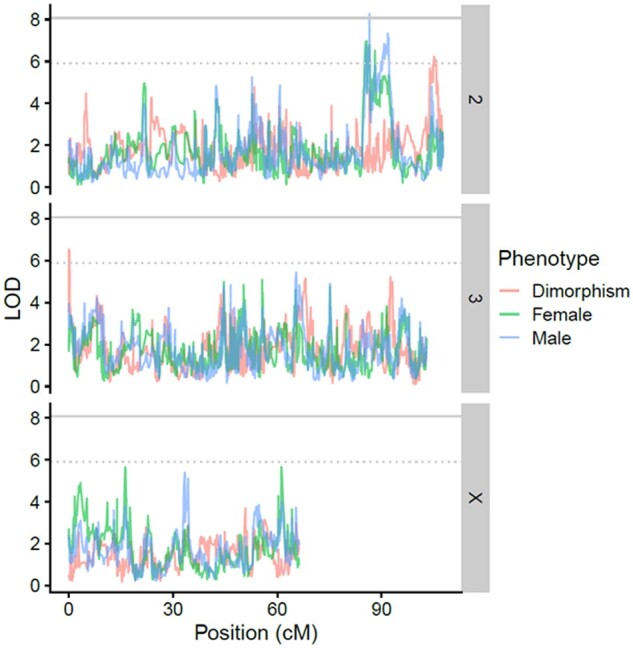
Positions of loci contributing to variation in immune defense in the QTL mapping study. The LOD score is plotted for each genomic position across chromosomes 2, 3, and X. The solid gray line is the 5% FDR and FWER (they are very close to each other). The dotted gray line is the 50% FDR. The QTL Mapping was done separately for males (blue), females (green), and the sexual dimorphism (red).

The family-wise significance threshold for QTL was a LOD score of 8.07, which was similar to the 5% FDR threshold (8.1), and using either yielded the same set of QTL. We identified one significant QTL using these thresholds from the male data (Figure 2). Supplementary Figure S3 shows the means for each set of RILs that have the different haplotypes at this significant QTL. This QTL explained 12% of the variance in male survival, however, given our sample size, this estimate is likely inflated (King and Long 2017). We additionally considered a more liberal FDR of 50%. At this lower threshold, we identified three QTL peaks (one peak for males, one for females, and one for the dimorphism) (Figure 2). The precise coordinates of these peaks are shown in Supplementary Table S2.

Among 297 Recombinant Inbred Lines from the Drosophila Synthetic Population Resource, we found vast diversity for 10-day survival post inoculation with the fungus *B. bassiana*, ranging from 0 flies surviving to 100% of flies surviving the infection. Our QTL mapping approach identified 451 genes (Supplementary Table S3) which we explored in Flybase (www.flybase.org; last accessed 9/18/21) for their functions. Specifically, one peak at a 5% FDR corresponded to 28 genes, including *Jabba*, known to be involved in bacterial defense, genes with functions in the endomembrane system (*Vps51*, *CG18609*, *CB17821*, and *Mctp*) and genes involved in oxidoreductase activity (*Cyp12b2*, *CG15093*, and *MFS14*). Three peaks at a 50% FDR included an additional 423 genes. These included 21 genes involved in the endomembrane system, including ones known to have immune functions (*e.g.*, *Snap29 and gbb*). Also implicated was the Bomanin 55C gene cluster, which has been shown to be induced by *B. bassiana* infection (De Gregorio *et al.* 2001). Other genes with pre-determined functions in immunity included *18w*, *GNBP-like3*, *DMAP1*, *imd*, *Nxt1*, and *St3*. The JNK, MAPK, and JAK/STAT pathways were also implicated through several genes, specifically *slim*, *apt*, *sigmar*, *ken*, *enok*, and *slbo*. Additionally, sensory perception of smell may be involved in defense against fungal infection; our study implicated odorant binding proteins (*Obp*) which assist in the sensory perception of smell (Rollmann *et al.* 2010) and *EbpIII* involved in odor recognition.

### Experimental evolution and resequencing

**Figure 3:**
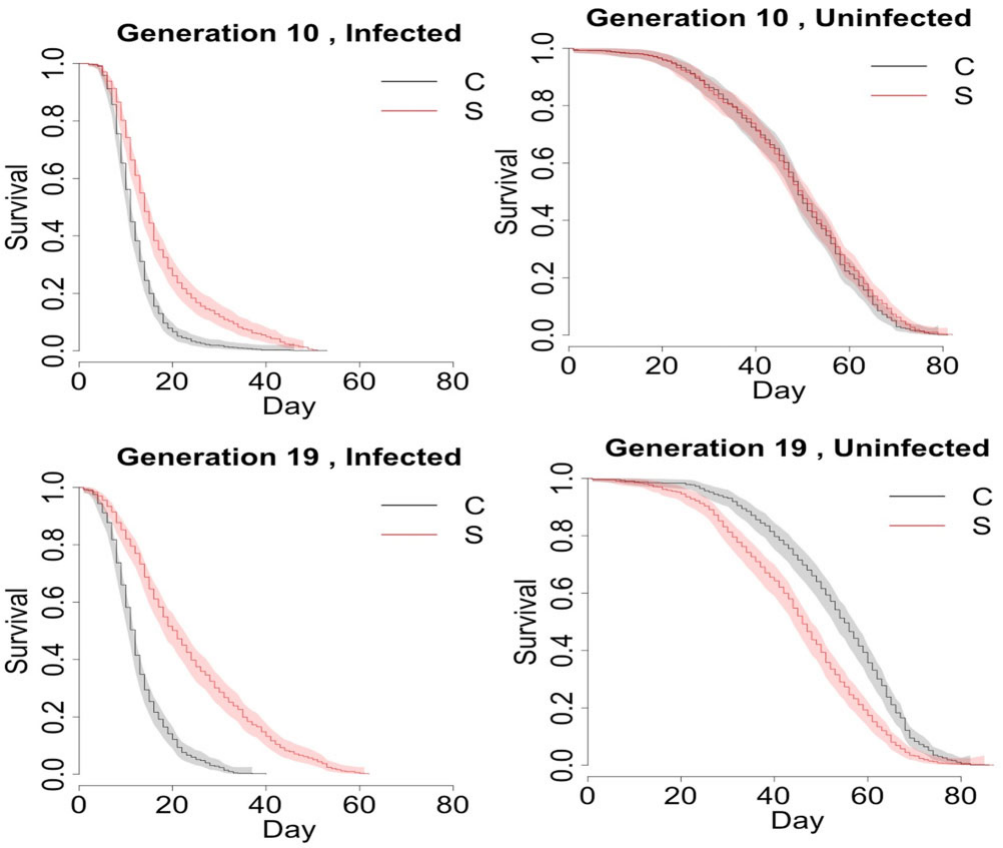
Survival of selected and control populations with and without infection with *B. bassiana* ARSEF 12460 at generations 10 and 19. The Kaplan-Meier estimate of the survival function is shown for immunity-selected S_1–4_ (red) and control C_1–4_ (black) populations across different combinations of infection status (columns; infection with *B. bassiana* ARSEF 12460) and generation (rows). The shaded area represents 95% confidence intervals. After 10 generations of selection for defense against the fungal entomopathogen *B. bassiana* ARSEF 12460, the S populations had higher survival compared to the C populations when infected with *B. bassiana* ARSEF 12460. After 19 generations of selection, the difference between infected S and C populations was even more pronounced. When the S and C populations were not infected, they did not differ in survival at generation 10. But at generation 19, the S populations survived worse compared to the C populations when uninfected, suggesting that their improved immune defense came at a trade-off with longevity in the absence of infection.

The survival differences in selected and control populations with and without infection with *B. bassiana* ARSEF 12460 are summarized in Table 1. The table shows the summary fit of the Cox Proportional Hazards model (see *Materials and Methods* model 1; Schoenfeld residual plots, not shown, confirmed the validity of proportional hazard assumptions). Selection for defense against *B. bassiana* ARSEF 12460 resulted in improved survival of infection, with selected populations living nearly twice as long when infected compared to control populations (Figure 3, Table 2). But this improved survival of infection came at a cost to survival under pathogen-free conditions, such that populations that evolved better immune defense had reduced lifespan than controls when uninfected (Figure 3, Table 2). Notably, the outbred populations in this study did not show sexual dimorphism in survival without infection, nor when infected with *B. bassiana* ARSEF 12460. We compared the different groups’ survivals by estimating the hazard ratio, which is a statistical measure of relative chances of death at all ages. For example, in Table 2, the estimated hazard ratio of 0.46 for infected S *vs* C at generation 10 means that the relative instantaneous probability of death for an infected selected fly was 46% of the instantaneous probability of death for an infected control fly, and the *P*-value for this comparison was *P* < 0.00001, implying significant divergence between survival of infection in the S and C populations. In other words, after just 10 generations, selection had resulted in substantially better survival of infection in the S populations. This difference increased by generation 19, such that at the end of the experiment, the instantaneous probability of death for an infected fly in the S populations was only 27% of the probability of death for an infected fly in the C populations (*P* < 0.00001) as estimated from a Cox Proportional Hazard Model. The hazard ratio (S *vs* C) among infected flies in generation 10 was significantly different from that of infected flies in generation 19 (the *P*-value of the test H_0_: HR_Gen10_ = HR_Gen19_ for infected groups was 0.00021), suggesting that between generations 10 and 19, the S populations continued to evolve improved survival of infection compared to the control populations.

**Table 1:** Analysis of survival differences in selected and control populations with and without infection with *B. bassiana* ARSEF 12460 at generations 10 and 19

	coef	exp(coef)	se(coef)	z	Pr(>|z|)
generation 19	−0.341	0.711	0.049	−6.982	2.91ε−12 ***
population S	−0.086	0.918	0.049	−1.760	0.078
infection	3.061	21.347	0.055	55.358	<2ε−16 ***
generation 19: population S	0.560	1.751	0.069	8.117	4.44ε−16 ***
generation 19: infection	0.240	1.272	0.067	3.582	0.000341 ***
population S: infection	−0.682	0.506	0.068	−9.979	<2ε−16 ***
generation 19: population S: infection	−1.113	0.328	0.097	−11.516	<2ε−16 ***

Survival was analyzed with model 1: coxph = P + G + D + P*G + P*D + G*D + P*G* where G represents generation (10 and 19), P represents populations (C and S), and D represents the infection status (uninfected and infected with 10^4^ spores/mm^2^). *N* = 7007.

**Table 2:** Hazard ratios from the Cox Proportional Hazard Model comparing selected (S) and control (C) populations across generation and infection status with *B. bassiana* ARSEF 12460

	Generation 10		Generation 19	
	Infected	Uninfected	Infected	Uninfected
Hazard ratio (S vs. C)	0.46	0.92	0.27	1.61
p-value	<0.00001	0.12	<0.00001	<0.00002

The reported *P*-values test the hypothesis H_0_: HR = 1, or no difference in hazard between the S and C populations.

Interestingly, after 19 generations of selection, the instantaneous probability of death for an uninfected fly in the S populations was 161% of the instantaneous probability of death of an uninfected fly in C populations (Table 2; *P* < 0.0002), meaning that populations that evolved improved immune defense paid the cost of reduced longevity in the absence of infection. This trade-off was not yet present at generation 10 (Table 2; *P* = 0.12). Thus, among uninfected flies, the hazard ratio (S *vs* C) was lower at generation 10 than at generation 19 (the *P*-value of the test H_0_: HR_Gen10_ = HR_Gen19_ for uninfected groups is 0.00083).

**Figure 4:**
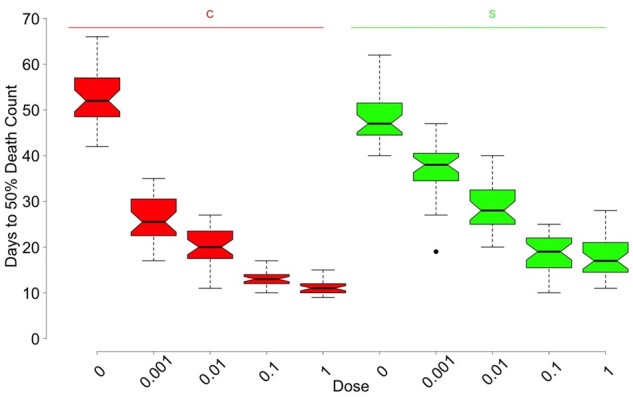
The median lethal time in days, or LT50, categorized by population type and infection dose. Data is combined across the replicate populations within each population type. Within each color, the 5 boxplots represent the 5 doses: 0, 0.001, 0.01, 0.1, and 1 (proportion relative to full dose of 10^4^ spores per mm^2^ of *B. bassiana* ARSEF 12460). LT50 was significantly affected by population (C *vs* S, *P* < 0.0001), generation (*P* = 0.036), and dose (*P* < 0.0001), but not by sex (Supplementary Table S4, Supplementary Figure S4).

We investigated the effect of population (S *vs* C), sex, generation (10 *vs* 19), and dose on LT_50_ with an ANOVA model (Supplementary Table S4 and Figure S4). The LT_50_ increased with decreasing dose in every subgroup, but the rate of increase varied among subgroups (Supplementary Figure S4). A simplified summary of the LT_50_ comparison between populations (S *vs* C) for different doses is visualized in Figure 4, where we see a drop in LT_50_ for C populations from the uninfected (dose 0) group to lowest infection dose at 0.001, which is more drastic than the same drop in the S populations. The decrease in LT_50_ is more gradual in the S populations compared to C populations. This implies that not only did the S populations evolve increased resistance to infection, but they are also more robust to escalating dose of the pathogen. LT50 was slightly higher (by a factor of 10%) in generation 19 compared to generation 10. Males and females did not differ in LT_50_ in either the S or C populations, nor when all data were combined (Supplementary Table S4 and Figure S4). Furthermore, once we adjust for the effect of population, generation, and dose, there is still only weak evidence of a difference between males and females.

#### Defense against B. bassiana GHA

We tested whether selection for defense against *B. bassiana* ARSEF 12460 also conferred resistance to a second *B. bassiana* strain, GHA. The summary fit for the Cox Proportional Hazards model (see Methods model 2) is presented in Supplementary Table S5. All four replicate populations that were selected for resistance against *B. bassiana* ARSEF 12460 also became more resistant to a second fungal strain, GHA (Supplementary Figure S5 and Table S6). The magnitude of the S *vs* C difference in survival after infection with GHA depended on both sex and generation (Supplementary Table S5). For males, at generation 10, the instantaneous probability of death for an S fly was 62% of the probability of death for a C fly (*P* = 0.0002) and by generation 19, the S males were surviving even better after infection, with an instantaneous probability of death of 44% compared to the C flies (*P* < 0.0001). In females, the difference between C and S in post-infection survival was not significant at generation 10, with the probability of death of an S female being 82% of that for a C female (*P* = 0.0243) but became significant by generation 19 with 45% probability of death of S *vs* C (*P* < 0.0001). This suggests that male flies evolved cross resistance to GHA faster than female flies. While overall survival after infection with GHA was higher in S flies than C flies at both generations 10 and 19 (Supplementary Table S6), the magnitude of this difference was larger in generation 19 (Supplementary Table S5).

Further supporting the sex differences in response to selection, for infection with GHA, we saw sexual dimorphism in the hazard ratio at generation 10 (H_0_: HR_male, Gen10_ = HR_female, Ge10_, *P*-value = 0.0256) with males surviving better than females, but not at generation 19 (H_0_: HR_male, Gen19_ = HR_female, Ge19_, *P* = 0.7134). This difference in sexual dimorphism across generations can also be noted from the sex-by-population-by-generation interaction (*P* < 0.0001; Supplementary Table S5). Moreover, for both sexes, the hazard ratio (S to C) in generation 10 is statistically higher than that of generation 19 (H_0_: HR_male, Gen10_ = HR_male, Ge19_, *P* = 0.0066, and H_0_ HR_female, Gen10_ = HR_female, Ge19_, *P* = 0.0004). Selection for survival of ARSEF 12460 resulted in improved defense against GHA, but unlike with ARSEF 12460, defense against GHA was sexually dimorphic at generation 10.

#### Defense against bacterial pathogens

Unlike with fungal infection, no difference was observed between S and C populations for resistance to bacterial infections with *E. faecalis and P. rettgeri* after 10 or 19 generations of selection (Supplementary Figure S6). There was also no difference between males and females (Supplementary Figure S6). Flies infected with either bacterium were much more likely to die than uninfected (sterile pricked) flies at both generations 10 and 19 (log-rank tests *P* < 0.0001). However, the likelihood of death was the same between S and C populations regardless of sex or generation (log-rank tests *P* > 0.05).

#### SNP variation

While we observe a number of chromosomal regions with notable depressions in heterozygosity in the P, C, and S populations, we do not see a dramatic loss in genetic variation in the C and S populations relative to the ancestral P population (Supplementary Figure S7). Heterozygosity here is a quantification of population diversity, thus levels of variation are very similar in the C and S populations compared to the ancestral P population. This pattern is largely robust to changes in window size (Supplementary Figures S8 and S9). However, we do find that depressions in heterozygosity become more pronounced as window size is reduced. We find that mean heterozygosity at polymorphic sites in the ancestral P population is 0.24, and ranges from 0.23 to 0.24 in the C and S populations (Supplementary Table S1). We do not find any significant difference in mean heterozygosity between the C and S groups (*t*-test *P*-value = 0.65). Among the C populations, we find that mean genome-wide *F_ST_* is 0.04. Mean genome-wide *F_ST_* is also 0.04 among the S group. In both cases, this suggests a high degree of similarity between replicates of a given group. The fact that levels of *F_ST_* are the same in each group is also consistent with the duration of the experiment (*i.e.*, there was not sufficient time for drift to produce high levels of divergence between replicates of a given group).

We examined sites that were fixed in all S populations at the end of the experiment but that remained polymorphic in the P and C populations. There were ∼4000 such sites distributed across chromosomes X, 2, and 3 (Supplementary Table S7). In about 98% of these instances, the frequency of the allele fixed in the S pops was ≥ 0.8 in the ancestral population. The lowest frequency we see in the ancestral population across all of these cases is 0.65. The SNP frequencies in the control populations at these ∼4000 sites follow the expectations from drift and have slight deviations from the ancestral frequencies. For comparison, we examined sites fixed in the C populations at the end of the experiment that were polymorphic in the S and P populations, which resulted in ∼1000 sites (Supplementary Table S8). These ∼1000 sites had high (>0.8) frequencies in the ancestral populations.

#### SNP differentiation

**Figure 5:**
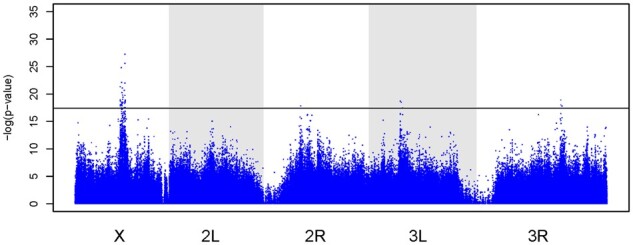
SNP frequency differentiation among selected (S) and control (C) populations analyzed with CMH tests. The -log(*P*-values) from CMH tests comparing SNP frequencies in the S and C populations are shown along all major chromosome arms. The black line indicates significance threshold derived from drift simulations.

SNPs and corresponding *P*-values from the CMH and GLM analyses are shown in Supplementary Table S9. Our CMH tests (Vlachos *et al.* 2019) comparing SNP frequencies in the C and S populations identified a total of 45 significantly differentiated SNPs across the major chromosome arms (Supplementary Table S10). The majority of these sites were found on the X chromosome, while the remaining were split between 3R, 3 L, and 2R. Mean SNP frequencies trajectories for C and S populations of the most significant SNPs in each region that crosses our significance threshold in Figure 5 are shown in Supplementary Figure S11.

In our quasi-binomial GLM results, no significantly differentiated sites were detected after the Bonferroni correction (Supplementary Figure S11A) was applied, or when the less stringent q-value approach was used to correct for multiple comparisons (Supplementary Figure S11B). Wiberg *et al.* (2017) report that the quasibinomial GLM approach has lower false positive and higher true positive rates than the CMH tests. However, given our simulation-based approach for correcting for multiple comparisons when using the CMH test, we have a more stringent significance threshold than what was used in Wiberg *et al*. (2017). Comparing Q-Q plots for the two approaches (Supplementary Figure S12), the CMH test appears better suited for our data than the quasi-binomial GLM method (note the different y-axis ranges in the two Q-Q plots).

#### Genes

There were 294 genes under the four peaks that cross the significance threshold in Figure 5 (Supplementary Table S11). Of these, 23 were involved in the endomembrane system (Supplementary Table S11). Several genes identified are known to be involved in response to oxidative stress (Supplementary Table S11); we identified genes *TotA*, *TotB*, *TotC*, and *TotZ* which have been shown to respond to bacterial infection (Ekengren and Hultmark 2001). Other known immune defense genes were also implicated, such as *GNBP3*, *Takl1*, *PGRP-SA*, and *CG2247*. Several genes affecting sensory perception of smell and taste were also identified (*e.g.*, *Gr10b*, *Or10a*, *Ir11a*).

#### GO terms

Running our list of candidate sites from the CMH tests through Gowinda, a tool that allows for analysis of gene set enrichment, identified 132 significantly enriched GO terms, which include nested and overlapping annotations (Kofler and Schlötterer 2012). Discarding terms with GO categories containing fewer than 2 genes and correcting for hierarchical clustering using GO-Module (Yang *et al.* 2011) reduced this list to 29 enriched terms (Supplementary Table S12; see Supplementary Table S13 for genes associated with each term).

## Discussion

Immune defense is a genetically complex and ecologically important trait with high levels of inter-individual variation. While much is already known about *D. melanogaster* immune defense against bacterial pathogens (reviewed in Buchon *et al.* 2014), defense against fungal pathogens has not been studied with the same depth. Yet insect defense against fungal infection has implications for biological control of crop pests and disease vectors. Here, we applied two common approaches for mapping defense against a fungal pathogen to *D. melanogaster* genes, quantitative trait locus (QTL) mapping, and experimental E&R.

Sensory perception of taste and smell came up in both our QTL and E&R studies. Sensory receptors have been previously shown to be involved in innate immunity (reviewed in Lee and Cohen 2015). Likewise, oxidative stress response, implicated in both of our studies has previously been shown to be potentially related to antifungal mechanism of the innate immune system that generate reactive oxygen species (*e.g.*, Dantas *et al.* 2015). Both of our studies point to the importance of the endomembrane system in fungal immune defense. Golgi apparatus organization and function may be important in defense against fungal infection. The Golgi apparatus’ role in innate immunity is becoming more recognized, particularly as a signaling platform for facilitating immune defense pathways (Dobbs *et al.* 2015; Mukai *et al.* 2016; Chen and Chen 2018; Tao *et al.* 2020). GO term analysis of our E&R results also implicated the endomembrane system as well as other membrane functions. But GO term analysis as applied to E&R studies such as ours implicitly assumes that many functionally related genes will be selected in the same way. This assumption would only be true if the trait was determined by many functionally related genes that each contribute small effects to the adaptive phenotype. We do not necessarily expect this genetic architecture, and we consider this GO term analysis with some caution. If evolution of the trait is driven by a few genes with large allelic effects, then we might expect a lack of conspicuous GO enrichment, as we observe in our analysis.

The Bomanin 55C gene cluster implicated in the QTL analysis provides particularly strong candidates and these have been shown to be induced by *B. bassiana* infection (De Gregorio *et al.* 2001). Deletion of this cluster results in susceptibility to infection with fungi, although effects on resistance to *B. bassiana* were not directly tested (Clemmons *et al.* 2015; Lindsay *et al.* 2018). Also strong candidates are the *Turandot* (*Tot*) genes identified in our E&R study, which have been shown to be involved in the Drosophila response to stress, injury, and infection (*e.g.*, Ekengren and Hultmark 2001).

Comparing the specific genes identified in our E&R and QTL mapping approaches, there were no direct overlaps. This could be due to different starting genetic composition of the populations and low power in each independent study. It is also possible that in the E&R study there are unintended selection pressures acting on the populations. While both approaches resulted in candidate genes that we can follow up on in future studies, the lists of candidate genes were unique. Given the complexity of the anti-fungal immunity phenotype, it is likely to be highly multigenic, and genes involved in this phenotype are likely to be involved in gene by gene, and gene by environment interactions. Thus, the lack of overlap between our two gene lists is not an indication of a lack of replication of these genes. Instead, both gene lists can be considered for further studies.

Some genes previously known to affect immune defense (Buchon *et al.* 2014) are not implicated in our study. Experimentally induced mutations or gene knockdowns that reveal extreme phenotypes due to loss of gene function may not detect the complexity of gene regulation and interactions. From an evolutionary perspective, only a small subset of genes and processes that can be mutated to give a phenotype in the laboratory are expected to be responsible for segregating phenotypic variation in nature (Gruber *et al.* 2007). Furthermore, phenotypic variation can be shaped by genes that are not classically considered part of the immune system. Polymorphisms with smaller effect on immune defense may be identifiable in experimental evolution and resequencing studies, which can address the molecular architecture of adaptation. Previous experimental evolution and resequencing studies have not always implicated canonical genes that can affect a trait. When diverse base populations are used in thoughtfully designed selection experiments (recommendations for experimental design are offered by Kofler and Schlötterer 2014; Schlötterer *et al.* 2015), if known loci that affect a trait are not implicated, those loci are perhaps unlikely to be commonly involved in the trait in outbred populations where the loci are affected by forces of natural selection.

We found that *D. melanogaster* populations that were exposed to *B. bassiana* infection quickly evolved improved defense against this fungus. There is some evidence that *B. bassiana* may be unique in terms of insect potential to evolve resistance to the pathogen under selection pressure (Dubovskiy *et al.* 2013). In our study, improvement in defense evolved within 10 generations and defense continued to improve until the termination of the experiment at nineteen generations. Rapid evolution of immune defense is possible because of standing genetic variation for host resistance. In wild populations, *D. melanogaster* defense against *B. bassiana* varies regionally (Tinsley *et al.* 2006; Paparazzo *et al.* 2015). For many traits, short-term adaptation appears to result primarily from existing genetic variants (Burke *et al.* 2010; Graves *et al.* 2017), and we would expect the same in our experiment. We maximized genetic diversity in our starting population by sampling flies from disparate geographic regions (Early and Clark 2017).

The evolution of immune defense against a fungal pathogen came at a cost to uninfected longevity, suggesting an evolutionary trade-off with a genetic basis. Evolutionary trade-offs due to antagonistic pleiotropy may act to maintain genetic variation for life history traits and immune defense (Roff 2002; Schmid-Hempel 2003; McKean and Lazzaro 2011) and may constrain the evolution and maintenance of immune defense (Lazzaro and Little 2009). The large amount of variation for fungal and bacterial immune defense within natural populations suggests that this character commonly trades-off with other fitness components, and this has been experimentally observed in *Drosophila* (Kraaijeveld and Godfray 1997; Luong and Polak 2007; Vijendravarma *et al.* 2009; Ye *et al.* 2009). Such tradeoffs can result from genetic correlations between fitness traits and immune defense. In addition to antagonistic pleiotropy, genetic correlations among these traits may also result from linkage disequilibrium. There are high levels of inversions in some of the founder lines used in our study, but the extent to which the resulting linkage disequilibrium affected our observed phenotypes is unknown. Some studies do not find any cost to laboratory evolved immune defense (Faria *et al.* 2015; Penley *et al.* 2018). For example, evolved immune defense through three selection regimes, oral and systemic infection with *P. entomophila* and systemic infection with Drosophila C virus, did not trade-off against reproductive output, development time, stress resistance, and other fitness characters (Faria *et al.* 2015), although Faria *et al.* (2015) did not examine potential trade-offs with uninfected longevity. It is possible that the costly trade-offs appear most prominently when there is a sudden shift to very high defense. For example, the selection pressure in the Faria *et al.* (2015) study was much weaker than in our study, with 33% of their population surviving in the first generation and the percentage increasing in later generations due to adaptation. This idea is further supported by Duncan *et al.* (2011), who saw costs to the protozoan *Paramecium caudatum* that were selected for increased defense against the bacterial pathogen *Holospora undulata*. However, when selection was relaxed, the fitness was restored without completely losing the evolved resistance (Duncan *et al.* 2011). Therefore, there may be a threshold rate at which improved immune defense can evolve without noticeable fitness costs. This could explain why there were no fitness costs observed when *C. elegans* populations that were formerly selected for defense against *Serratia marcescens* maintained their increased resistance despite 16 generations of relaxed selection (Penley *et al.* 2018), or why no apparent fitness costs were seen when *D. melanogaster* were evolved for resistance to bacterial infection (Gupta *et al.* 2016).

The trade-off between fungal immune defense and uninfected longevity is not axiomatic. Indeed *D. melanogaster* populations that are experimentally evolved for resistance against other stressors, in particular starvation and desiccation, have increased longevity (Rose *et al.* 1992; Bubliy and Loeschcke 2005). The increased longevity that evolves alongside stress resistance is sometimes maintained even after stress resistance reverts back to ancestral levels after a period of relaxed selection, even when the relaxed selection results from shifts in allele frequencies rather than any compensatory mutations (Phillips *et al.* 2018). Presumably, living longer requires effective stress resistance, and perhaps also a strong immune defense. Resistance and tolerance of infection both decline with age (reviewed in Garschall and Flatt 2018) and susceptibility to infection increases with age (Kubiak and Tinsley 2017), thus it may be expected that increases in longevity should be correlated with improvements in immune defense. Indeed, this has been observed, such that experimental evolution for delayed reproduction, which increases longevity, improves immune defense in *D. melanogaster* (Fabian *et al.* 2018). It is also possible that the presence or absence of a trade-off between lifespan and immunity depends on specific environmental factors (*e.g.*, McKean et al. 2008).

In addition to trade-offs of immunity with fitness characters, another potential reason for populations maintaining variation in immune defense is that different genotypes are most resistant to specific pathogens, which would lead to trade-offs within the immune system for defense against different pathogens. Evolution of immune defense against one pathogen may trade-off with defense against a second pathogen. For example, *D. melanogaster* selected for resistance against bacteria paid a cost in the presence of viruses (Martins *et al.* 2013). We tested our experimentally evolved populations for cross resistance against other pathogens and found that evolution of immune defense against one strain of *B. bassiana* (ARSEF 12460) unsurprisingly also led to improved defense against a second strain of *B. bassiana* (GHA). If evolution of defense against *B. bassiana* was primarily through this humoral immune response, we may expect populations with increased defense against fungi to also have improved defense against Gram-positive *Enterococcus faecalis*, but we did not observe this. Evolved defense against fungus had no effect on defense against *E. faecalis*, nor against the Gram-negative bacterium *Providencia rettgeri*. Evolved defense may therefore be through other mechanisms than canonical immune defense pathways. For example, our analysis points to the importance of the endomembrane system function in defense against *B. bassiana*.

Unlike our study, Wang *et al.* (2017) found that in the Drosophila Genetics Reference Panel (DGRP), defense against the fungus *Metarhizium anisopliae* Ma549 was positively correlated with defense against the Gram-negative bacterium *Pseudomonas aeruginosa* Pa14. Yet no correlation was seen between resistance to enteric infection with *P. entomophila* and inoculation (by stabbing) with *Erwinia carotovora* (Sleiman *et al.* 2015). It appears that *D. melanogaster* adaptation to parasites depends in part on the infection route, such that selection by oral infection against *Pseudomonas entomophila* did not confer resistance against systemic infection and vice versa (Martins *et al.* 2013). In our study, the two fungi were introduced by spray onto the fly cuticle. After contact with the cuticle, the fungus germinates and the hyphae penetrate the cuticle, presumably at multiple locations on the cuticle, and grow in the hemocoel of the fly. Our bacterial infections were done by pin prick into the fly thorax, thus leading to a localized wound on the cuticle. These different infection routes may be another reason for why we see no cross-resistance of fungal resistant populations against bacterial pathogens. But even with similar pathogens and similar infection routes, the same *D. melano*gaster genotypes are not resistant to all bacterial pathogens (Lazzaro *et al.* 2006). Defense against pathogens likely involves many genes, and potentially some of these may confer pathogen-specific defense, while others may contribute to some general aspect of robustness.

It is worth noting that in this study we used larger population sizes than those commonly used in *D. melanogaster* experimental evolution studies, starting our selection protocol with 10,000 individuals in each replicate population. With large population sizes, four replicates per treatment, and nineteen generations of selection, we identified few candidate genes involved in immune defense against *B. bassiana*, despite the large phenotypic divergence between selected and control populations. This may be due to our study being underpowered by only having four replicate selected populations. While this level of replication is typical of existing E&R studies in *D. melanogaster*, some studies suggest that it may not be sufficient for detecting causal variants (Baldwin-Brown *et al.* 2014; Kofler and Schlötterer 2014). With current genomic tools, many more generations, and more replicates, may be needed to provide sufficient power to detect the many small-effect genes that are expected to confer immune defense.

Artificial selection experiments often display changes in SNP frequencies that appear to plateau before the end of the experiment, even as the phenotype continues to respond to selection. Parts *et al.* (2011) observed plateaus at intermediate allele frequencies in their yeast experimental evolution study, suggesting a reduction in selection coefficients. Such reductions in selection coefficients have been modeled by Illingworth *et al.* (2012), and plateaus in allele frequencies were also observed in *D. melanogaster* laboratory adaptation (Orozco-Terwengel *et al.* 2012). We observed 4000 sites across the entire genome that became fixed in the selected populations but were polymorphic in the ancestral and control populations. In comparison, only 1000 sites fixed in the control populations but remained polymorphic in the ancestral and selected populations. Given the large effective population sizes in the S and C populations in every generation (*N* > 1,000), the list of fixed sites in the S populations likely resulted from selection instead of drift, and may serve as candidate sites for immune defense variation.

In the E&R study and in the QTL Mapping study, we saw evidence of sexual dimorphism in immune defense. A better understanding of what leads to sexual dimorphism in immune defense may guide the use of *B. bassiana* in biological control efforts that may benefit from targeting female insects. Previous studies have suggested that *D. melanogaster* females are more susceptible to infection with *B. bassiana* infection compared to males (Taylor and Kimbrell 2007; Kubiak *and Tinsley* 2017; Shahrestani *et al.* 2018). Using the same *B. bassiana* ARSEF 12460 pathogen, we previously found that female flies were more susceptible to infection than male flies in inbred fly lines (Shahrestani *et al.* 2018), and this direction of sexual dimorphism was maintained whether the flies were sprayed or injected with the fungus, suggesting that grooming and barrier defenses were not fully responsible for sexual dimorphism in defense.

In our E&R study, there was sexual dimorphism in immune defense against *B. bassiana* only in the rate of evolution of cross-resistance to GHA, which evolved faster in males than females. It is unclear to us why cross-resistance should evolve faster in males. In our QTL mapping study, we found that the presence and direction of sexual dimorphism in immune defense is dependent on fly genotype. The host genetic factors that affect the direction and magnitude of sexual dimorphism in immune defense remain a topic to investigate. One common hypothesis for sexual dimorphism in immune defense is differential reproductive investment of males and females leading to different resource allocation (reviewed in Schwenke *et al.* 2016). We do not have data for the reproductive output of the RILs used in this study, but it would be interesting to compare fecundity with sexual dimorphism in immune defense. Given the variation in both direction and magnitude of the observed sexual dimorphism, it is possible that several mechanisms can be involved in this trait. Despite the sexual dimorphism in defense, there was a positive correlation in 10-day survival of infection of males and females among the RILs.

Overall, we have shown that immune defense against a fungal pathogen is highly variable in *D. melanogaster* derived from natural populations. This variability allows rapid adaption in response to experimental selection, albeit at a cost to uninfected longevity. The presence of such extensive naturally occurring genetic variation suggests considerable adaptive potential in nature, although perhaps buffered by costs and tradeoffs. Notably, the variation appears to be distributed among multiple genes with modest allelic effects and no clear enrichment of functional gene categories. Nevertheless, using two different experimental approaches, we have identified a set of potentially causal genes that may be promising candidates for future study.

## Data availability

Supplementary files have been added to figshare: https://doi.org/10.25387/g3.15141024.

These files include raw data for the RIL phenotypes (Supplementary File S1), phenotype data for the S and C populations (Supplementary Files S2−S4), SNP tables (Supplementary File S5), CMH and GLM results (Supplementary Table S9), major scripts, and simulation sync files and simulation results.

## Author contributions

P.S., B.P.L., and A.G.C. conceived of the experiment. P.S., K.G., P.C., G.M., Y.E., M.Z., S.R., and M.G. performed the experiments and collected all data. E.K., R.R., M.P., Z.G., M.R., and P.S. analyzed the data. P.S., M.P., M.T., M.R., A.G.C., and B.P.L. wrote the manuscript. B.P.L., A.G.C., J.V., S.W., and A.D.L. provided training, expertise, and resources.

## References

[jkab324-B1] Baldwin-Brown JG , Long AD, Thornton KR. 2014. The power to detect quantitative trait loci using resequenced, experimentally evolved populations of diploid, sexual organisms. Mol Biol Evol. 31:1040–1055. 24441104 10.1093/molbev/msu048 PMC3969567

[jkab324-B2] Barbarin AM , Jenkins NE, Rajotte EG, Thomas MB. 2012. A preliminary evaluation of the potential of *Beauveria bassiana* for bed bug control. J Invertebr Pathol. 111:82–85. 22555012 10.1016/j.jip.2012.04.009

[jkab324-B3] Bates DM , Maechler M, Bolker BM, Walker S. 2015. Fitting linear mixed‐effects models using lme4. J Stat Softw. 67:1–48.

[jkab324-B4] Begun DJ , Aquadro CF. 1993. African and North American populations of *Drosophila melanogaster* are very different at the DNA level. Nature. 365:548–550. 8413609 10.1038/365548a0

[jkab324-B5] Begun DJ , Aquadro CF. 1995. Molecular variation at the vermilion locus in geographically diverse populations of *Drosophila melanogaster* and *D. simulans*. Genetics. 140:1019–1032. 7672574 10.1093/genetics/140.3.1019 PMC1206658

[jkab324-B6] Benjamini Y , Hochberg Y. 1995. Controlling the false discovery rate: a practical and powerful approach to multiple testing. J R Stat Soc B Methodol. 57:289–300.

[jkab324-B7] Berriz GF , Beaver JE, Cenik C, Tasan M, Roth FP. 2009. Next generation software for functional trend analysis. Bioinformatics. 25:3043–3044. 19717575 10.1093/bioinformatics/btp498 PMC2800365

[jkab324-B8] Bochdanovits Z , Jong GD. 2003. Temperature dependence of fitness components in geographical populations of *Drosophila melanogaster*: changing the association between size and fitness. Biol J Linn Soc. 80:717–725.

[jkab324-B9] Broman KW , Sen S. 2009. A Guide to QTL Mapping with R/QTL. New York, NY: Springer-Verlag.

[jkab324-B10] Bubliy OA , Loeschcke V. 2005. Correlated responses to selection for stress resistance and longevity in a laboratory population of *Drosophila melanogaster*. J Evol Biol. 18:789–803. 16033550 10.1111/j.1420-9101.2005.00928.x

[jkab324-B11] Buchon N , Silverman N, Cherry S. 2014. Immunity in *Drosophila melanogaster*—from microbial recognition to whole-organism physiology. Nat Rev Immunol. 14:796–810. 25421701 10.1038/nri3763 PMC6190593

[jkab324-B12] Burke MK , Dunham JP, Shahrestani P, Thornton KR, Rose MR, et al 2010. Genome-wide analysis of a long-term evolution experiment with *Drosophila*. Nature. 467:587–590. 20844486 10.1038/nature09352

[jkab324-B13] Chen J , Chen ZJ. 2018. PtdIns4P on dispersed trans-Golgi network mediates NLRP3 inflammasome activation. Nature. 564:71–76. 30487600 10.1038/s41586-018-0761-3 PMC9402428

[jkab324-B14] Churchill GA , Doerge RW. 1994. Empirical threshold values for quantitative trait mapping. Genetics. 138:963–971. 7851788 10.1093/genetics/138.3.963 PMC1206241

[jkab324-B15] Clemmons AW , Lindsay SA, Wasserman SA. 2015. An effector peptide family required for Drosophila Toll-mediated immunity. PLoS Pathog. 11:e1004876. 25915418 10.1371/journal.ppat.1004876 PMC4411088

[jkab324-B16] Cox DR , Lewis PAW. 1972. Multivariate point processes. In Proceedings of 6th Berkeley Symposium Mathematical Statistical Probability. 3:401–448

[jkab324-B17] Dantas ADS , Day A, Ikeh M, Kos I, Achan B, et al 2015. Oxidative stress responses in the human fungal pathogen, *Candida albicans*. Biomolecules. 5:142–165. 25723552 10.3390/biom5010142 PMC4384116

[jkab324-B18] De Gregorio E , Spellman PT, Rubin GM, Lemaitre B. 2001. Genome-wide analysis of the Drosophila immune response by using oligonucleotide microarrays. Proc Natl Acad Sci USA. 98:12590–12595. 11606746 10.1073/pnas.221458698 PMC60098

[jkab324-B19] Dobbs N , Burnaevskiy N, Chen D, Gonugunta VK, Alto NM, et al 2015. STING activation by translocation from the ER is associated with infection and autoinflammatory disease. Cell Host Microbe. 18:157–168. 26235147 10.1016/j.chom.2015.07.001 PMC4537353

[jkab324-B20] Dubovskiy IM , Whitten MM, Yaroslavtseva ON, Greig C, Kryukov VY, et al 2013. Can insects develop resistance to insect pathogenic fungi? PLoS One. 8:e60248. 23560083 10.1371/journal.pone.0060248 PMC3613352

[jkab324-B21] Duncan AB , Fellous S, Kaltz O. 2011. Reverse evolution: selection against costly resistance in disease‐free microcosm populations of *Paramecium caudatum*. Evolution. 65:3462–3474. 22133218 10.1111/j.1558-5646.2011.01388.x

[jkab324-B101] Duxbury, E. M., Day, J. P., Vespasiani, D. M., Thüringer, Y., Tolosana, I., Smith, S. C., et al. (2019). Host-pathogen coevolution increases genetic variation in susceptibility to infection. Elife, 8:e46440. 10.7554/eLife.46440 PMC6491035 31038124

[jkab324-B22] Early AM , Clark AG. 2017. Genomic signatures of local adaptation in the *Drosophila* immune response. Fly (Austin). 11:277–283. 28586288 10.1080/19336934.2017.1337612 PMC5721942

[jkab324-B23] Ekengren S , Hultmark D. 2001. A family of Turandot-related genes in the humoral stress response of Drosophila. Biochem Biophys Res Commun. 284:998–1003. 11409894 10.1006/bbrc.2001.5067

[jkab324-B24] Fabian DK , Garschall K, Klepsatel P, Santos‐Matos G, Sucena E, et al 2018. Evolution of longevity improves immunity in Drosophila. Evol Lett. 2:567–579. 30564440 10.1002/evl3.89 PMC6292704

[jkab324-B25] Faria VG , Martins NE, Paulo T, Teixeira L, Sucena É, et al 2015. Evolution of *Drosophila* resistance against different pathogens and infection routes entails no detectable maintenance costs. Evolution. 69:2799–2809. 26496003 10.1111/evo.12782

[jkab324-B26] Feder AF , Petrov DA, Bergland AO. 2012. LDx: estimation of linkage disequilibrium from high-throughput pooled resequencing data. PLoS One. 7:e48588. 23152785 10.1371/journal.pone.0048588 PMC3494690

[jkab324-B27] Fiston-Lavier AS , Singh ND, Lipatov M, Petrov DA. 2010. *Drosophila melanogaster* recombination rate calculator. Gene. 463:18–20. 20452408 10.1016/j.gene.2010.04.015

[jkab324-B28] García-Munguía AM , Garza-Hernández JA, Rebollar-Tellez EA, Rodríguez-Pérez MA, Reyes-Villanueva F. 2011. Transmission of *Beauveria bassiana* from male to female *Aedes aegypti* mosquitoes. Parasit Vectors. 4:24. 21352560 10.1186/1756-3305-4-24 PMC3051917

[jkab324-B29] Garschall K , Flatt T. 2018. The interplay between immunity and aging in Drosophila. F1000Res. 7:160. 29487742 10.12688/f1000research.13117.1 PMC5806056

[jkab324-B30] Graves JL Jr , Hertweck KL, Phillips MA, Han MV, Cabral LG, et al 2017. Genomics of parallel experimental evolution in *Drosophila*. Mol Biol Evol. 34:831–842. 28087779 10.1093/molbev/msw282 PMC5400383

[jkab324-B31] Greenberg AJ , Hackett SR, Harshman LG, Clark AG. 2010. A hierarchical Bayesian model for a novel sparse partial diallel crossing design. Genetics. 185:361–373. 20157001 10.1534/genetics.110.115055 PMC2870970

[jkab324-B33] Gruber JD , Genissel A, Macdonald SJ, Long AD. 2007. How repeatable are associations between polymorphisms in achaete-scute and bristle number variation in Drosophila? Genetics. 175:1987–1997. 17277365 10.1534/genetics.106.067108 PMC1855119

[jkab324-B34] Gupta V , Venkatesan S, Chatterjee M, Syed ZA, Nivsarkar V, et al 2016. No apparent cost of evolved immune response in *Drosophila melanogaster*. Evolution. 70:934–943. 26932243 10.1111/evo.12896

[jkab324-B36] Hedrick P. 2011. Genetics of Populations. Jones & Bartlett Learning.

[jkab324-B37] Illingworth CJ , Parts L, Schiffels S, Liti G, Mustonen V. 2012. Quantifying selection acting on a complex trait using allele frequency time series data. Mol Biol Evol. 29:1187–1197. 22114362 10.1093/molbev/msr289 PMC3731369

[jkab324-B39] Jónás A , Taus T, Kosiol C, Schlötterer C, Futschik A. 2016. Estimating the effective population size from temporal allele frequency changes in experimental evolution. Genetics. 204:723–735. 27542959 10.1534/genetics.116.191197 PMC5068858

[jkab324-B40] Kaplan EL , Meier P. 1958. Nonparametric estimation from incomplete observations. JASA. 53:457–481.

[jkab324-B41] Khalil S , Jacobson E, Chambers MC, Lazzaro BP. 2015. Systemic bacterial infection and immune defense phenotypes in *Drosophila melanogaster*. J Vis Exp. 99:e52613. 10.3791/52613 PMC4542538 25992475

[jkab324-B42] King EG , Macdonald SJ, Long AD. 2012a. Properties and power of the Drosophila Synthetic Population Resource for the routine dissection of complex traits. Genetics. 191:935–949. 22505626 10.1534/genetics.112.138537 PMC3389985

[jkab324-B43] King EG , Merkes CM, McNeil CL, Hoofer SR, Sen S, et al 2012b. Genetic dissection of a model complex trait using the Drosophila Synthetic Population Resource. Genome Res. 22:1558–1566. 22496517 10.1101/gr.134031.111 PMC3409269

[jkab324-B44] King EG , Long AD. 2017. The Beavis effect in next-generation mapping panels in *Drosophila melanogaster*. G3 (Bethesda). 7:1643–1652. 28592647 10.1534/g3.117.041426 PMC5473746

[jkab324-B45] Kislukhin G , King EG, Walters KN, Macdonald SJ, Long AD. 2013. The genetic architecture of methotrexate toxicity is similar in *Drosophila melanogaster* and humans. G3 (Bethesda). 3:1301–1310. 23733889 10.1534/g3.113.006619 PMC3737169

[jkab324-B46] Kofler R , Pandey RV, Schlötterer C. 2011. PoPoolation2: identifying differentiation between populations using sequencing of pooled DNA samples (Pool-Seq). Bioinformatics. 27:3435–3436. 22025480 10.1093/bioinformatics/btr589 PMC3232374

[jkab324-B47] Kofler R , Schlötterer C. 2012. Gowinda: unbiased analysis of gene set enrichment for genome-wide association studies. Bioinformatics. 28:2084–2085. 22635606 10.1093/bioinformatics/bts315 PMC3400962

[jkab324-B48] Kofler R , Schlötterer C. 2014. A guide for the design of evolve and resequencing studies. Mol Biol Evol. 31:474–483. 24214537 10.1093/molbev/mst221 PMC3907048

[jkab324-B49] Kolaczkowski B , Kern AD, Holloway AK, Begun DJ. 2011. Genomic differentiation between temperate and tropical Australian populations of *Drosophila melanogaster*. Genetics. 187:245–260. 21059887 10.1534/genetics.110.123059 PMC3018305

[jkab324-B50] Kraaijeveld AR , Godfray HCJ. 1997. Trade-off between parasitoid resistance and larval competitive ability in *Drosophila melanogaster*. Nature. 389:278–280. 9305840 10.1038/38483

[jkab324-B51] Kubiak M , Tinsley MC. 2017. Sex-specific routes to immune senescence in *Drosophila melanogaster*. Sci Rep. 7:10417. 28874758 10.1038/s41598-017-11021-6 PMC5585412

[jkab324-B52] Lazzaro BP , Little TJ. 2009. Immunity in a variable world. Philos Trans R Soc Lond B Biol Sci. 364:15–26. 18926975 10.1098/rstb.2008.0141 PMC2666692

[jkab324-B53] Lazzaro BP , Sackton TB, Clark AG. 2006. Genetic variation in *Drosophila melanogaster* resistance to infection: a comparison across bacteria. Genetics. 174:1539–1554. 16888344 10.1534/genetics.105.054593 PMC1667071

[jkab324-B54] Lee RJ , Cohen NA. 2015. Taste receptors in innate immunity. Cell Mol Life Sci. 72:217–236. 25323130 10.1007/s00018-014-1736-7 PMC4286424

[jkab324-B55] Li H , Durbin R. 2009. Fast and accurate short read alignment with Burrows–Wheeler transform. Bioinformatics. 25:1754–1760. 19451168 10.1093/bioinformatics/btp324 PMC2705234

[jkab324-B56] Li H , Handsaker B, Wysoker A, Fennell T, Ruan J, et al; 1000 Genome Project Data Processing Subgroup, 2009. The sequence alignment/map format and SAMtools. Bioinformatics. 25:2078–2079. 19505943 10.1093/bioinformatics/btp352 PMC2723002

[jkab324-B57] Li Z , Alves SB, Roberts DW, Fan M, Delalibera I, et al 2010. Biological control of insects in Brazil and China: history, current programs and reasons for their successes using entomopathogenic fungi. Bioconrol Sci Technol. 20:117–136.

[jkab324-B58] Lindsay SA , Lin SJ, Wasserman SA. 2018. Short-Form bomanins mediate humoral immunity in *Drosophila*. J Innate Immun. 10:306–314. 29920489 10.1159/000489831 PMC6158068

[jkab324-B59] Long AD , Macdonald SJ, King EG. 2014. Dissecting complex traits using the Drosophila Synthetic Population Resource. Trends Genet. 30:488–495. 25175100 10.1016/j.tig.2014.07.009 PMC4253597

[jkab324-B61] Luong LT , Polak M. 2007. Costs of resistance in the Drosophila–Macrocheles system: a negative genetic correlation between ectoparasite resistance and reproduction. Evolution. 61:1391–1402. 17542848 10.1111/j.1558-5646.2007.00116.x

[jkab324-B62] Macdonald SJ , Long AD. 2007. Joint estimates of quantitative trait locus effect and frequency using synthetic recombinant populations of *Drosophila melanogaster*. Genetics. 176:1261–1281. 17435224 10.1534/genetics.106.069641 PMC1894589

[jkab324-B63] Martins NE , Faria VG, Teixeira L, Magalhães S, Sucena É. 2013. Host adaptation is contingent upon the infection route taken by pathogens. PLoS Pathog. 9:e1003601. 24086131 10.1371/journal.ppat.1003601 PMC3784483

[jkab324-B64] McKean KA , Yourth CP, Lazzaro BP, Clark AG. 2008. The evolutionary costs of immunological maintenance and deployment. BMC Evol Biol. 8:76. 18315877 10.1186/1471-2148-8-76 PMC2292698

[jkab324-B65] McKean KA , Lazzaro BP. 2011. The costs of immunity and the evolution of immunological defense mechanisms. In: T. Flatt, A. Heyland, editors. Mechanisms of Life History Evolution, Oxford: Oxford University Press. 299–310.

[jkab324-B66] Mukai K , Konno H, Akiba T, Uemura T, Waguri S, et al 2016. Activation of STING requires palmitoylation at the Golgi. Nat Commun. 7:1–10. 10.1038/ncomms11932 PMC4919521 27324217

[jkab324-B67] Najarro MA , Hackett JL, Macdonald SJ. 2017. Loci contributing to boric acid toxicity in two reference populations of *Drosophila melanogaster*. G3 (Bethesda). 7:1631–1641. 28592646 10.1534/g3.117.041418 PMC5473745

[jkab324-B68] Najarro MA , Hackett JL, Smith BR, Highfill CA, King EG, et al 2015. Identifying loci contributing to natural variation in xenobiotic resistance in *Drosophila*. PLoS Genet. 11:e1005663. 26619284 10.1371/journal.pgen.1005663 PMC4664282

[jkab324-B69] Orozco‐Terwengel P , Kapun M, Nolte V, Kofler R, Flatt T, et al 2012. Adaptation of *Drosophila* to a novel laboratory environment reveals temporally heterogeneous trajectories of selected alleles. Mol Ecol. 21:4931–4941. 22726122 10.1111/j.1365-294X.2012.05673.x PMC3533796

[jkab324-B70] Paparazzo F , Tellier A, Stephan W, Hutter S. 2015. Survival rate and transcriptional response upon infection with the generalist parasite *Beauveria bassiana* in a world-wide sample of *Drosophila melanogaster*. PLoS One. 10:e0132129. 26154519 10.1371/journal.pone.0132129 PMC4495925

[jkab324-B71] Parts L , Cubillos F, Warringer J, Jain K, Salinas F, et al 2011. Revealing the genetic structure of a trait by sequencing a population under selection. Genome Res. 21:1131–1138. 21422276 10.1101/gr.116731.110 PMC3129255

[jkab324-B72] Penley MJ , Greenberg AB, Khalid A, Namburar SR, Morran LT. 2018. No measurable fitness cost to experimentally evolved host defense in the *Caenorhabditis elegans*–*Serratia marcescens* host–parasite system. J Evol Biol. 31:1976–1981. 30187979 10.1111/jeb.13372

[jkab324-B73] Pinheiro J , Bates D, DebRoy S, Sarkar D. 2012. 2020 R Development Core Team. nlme: Linear and nonlinear mixed effects models. http://CRAN.R-project.org/package=nlme*. R package version*, 3–1.

[jkab324-B100] Phillips, M. A., Rutledge, G. A., Kezos, J. N., Greenspan, Z. S., Talbott, A., Matty, S., et al. (2018). Effects of evolutionary history on genome wide and phenotypic convergence in Drosophila populations. BMC genomics, 19:1–17. 10.1186/s12864-018-5118-7 PMC6180417 30305018

[jkab324-B74] R Core Team 2017. R: A Language and Environment for Statistical Computing. Vienna, Austria: R Foundation for Statistical Computing. https://www.R-project.org/.

[jkab324-B75] Roff DA. 1997. Evolutionary Quantitative Genetics. Springer US.

[jkab324-B76] Roff DA. 2002. *Life history evolution* (No. 576.54 R6).

[jkab324-B77] Rollmann SM , Wang P, Date P, West SA, Mackay TF, et al 2010. Odorant receptor polymorphisms and natural variation in olfactory behavior in *Drosophila melanogaster*. Genetics. 186:687–697. 20628035 10.1534/genetics.110.119446 PMC2954467

[jkab324-B78] Rose MR , Vu LN, Park SU, Graves JL. Jr, 1992. Selection on stress resistance increases longevity in *Drosophila melanogaster*. Exp Gerontol. 27:241–250. 1521597 10.1016/0531-5565(92)90048-5

[jkab324-B81] Schlötterer C , Kofler R, Versace E, Tobler R, Franssen SU. 2015. Combining experimental evolution with next-generation sequencing: a powerful tool to study adaptation from standing genetic variation. Heredity (Edinb). 114:431–440. 25269380 10.1038/hdy.2014.86 PMC4815507

[jkab324-B82] Schmid-Hempel P. 2003. Variation in immune defence as a question of evolutionary ecology. Proc Biol Sci. 270:357–366. 12639314 10.1098/rspb.2002.2265 PMC1691258

[jkab324-B83] Schwenke RA , Lazzaro BP, Wolfner MF. 2016. Reproduction–immunity trade-offs in insects. Annu Rev Entomol. 61:239–256. 26667271 10.1146/annurev-ento-010715-023924 PMC5231921

[jkab324-B84] Shahrestani P , Chambers M, Vandenberg J, Garcia K, Malaret G, et al 2018. Sexual dimorphism in *Drosophila melanogaster* survival of *Beauveria bassiana* infection depends on core immune signaling. Sci Rep. 8:12501–12509. 30131599 10.1038/s41598-018-30527-1 PMC6104035

[jkab324-B85] Sleiman MSB , Osman D, Massouras A, Hoffmann AA, Lemaitre B, et al 2015. Genetic, molecular and physiological basis of variation in Drosophila gut immunocompetence. Nat Commun. 6:7829. 26213329 10.1038/ncomms8829 PMC4525169

[jkab324-B86] Storey JD , Bass A, Dabney A, Robinson D. 2017. q-value: Q-value estimation for false discovery rate control. R package version 2.2.2. 2015. http://github.com/jdstorey/qvalue.

[jkab324-B87] Storey JD , Tibshirani R. 2003. Statistical significance for genomewide studies. Proc Natl Acad Sci USA. 100:9440–9445. 12883005 10.1073/pnas.1530509100 PMC170937

[jkab324-B88] Tao Y , Yang Y, Zhou R, Gong T. 2020. Golgi apparatus: an emerging platform for innate immunity. Trends Cell Biol. 30:467–477. 32413316 10.1016/j.tcb.2020.02.008

[jkab324-B89] Taylor K , Kimbrell D. 2007. Host immune response and differential survival of the sexes in Drosophila. Fly (Austin). 1:197–204. 18820477 10.4161/fly.5082

[jkab324-B90] Tinsley MC , Blanford S, Jiggins FM. 2006. Genetic variation in *Drosophila melanogaster* pathogen susceptibility. Parasitology. 132:767–773. 16497252 10.1017/S0031182006009929 PMC1847563

[jkab324-B91] Ugine TA , Wraight SP, Brownbridge M, Sanderson JP. 2005. Development of a novel bioassay for estimation of median lethal concentrations (LC50) and doses (LD50) of the entomopathogenic fungus *Beauveria bassiana*, against western flower thrips, *Frankliniella occidentalis*. J Invertebr Pathol. 89:210–218. 16039665 10.1016/j.jip.2005.05.010

[jkab324-B92] Valero‐Jiménez CA , van Kan JA, Koenraadt CJ, Zwaan BJ, Schoustra SE. 2017. Experimental evolution to increase the efficacy of the entomopathogenic fungus *Beauveria bassiana* against malaria mosquitoes: effects on mycelial growth and virulence. Evol Appl. 10:433–443. 28515777 10.1111/eva.12451 PMC5427670

[jkab324-B93] Vandenberg JD. 1996. Standardized bioassay and screening of *Beauveria bassiana* and *Paecilomyces fumosoroseus* against the Russian wheat aphid (Homoptera: Aphididae). J Econ Entomol. 89:1418–1423.

[jkab324-B94] Vijendravarma RK , Kraaijeveld AR, Godfray HCJ. 2009. Experimental evolution shows *Drosophila melanogaster* resistance to a microsporidian pathogen has fitness costs. Evolution. 63:104–114. 18786186 10.1111/j.1558-5646.2008.00516.x

[jkab324-B95] Vlachos C , Burny C, Pelizzola M, Borges R, Futschik A, et al 2019. Benchmarking software tools for detecting and quantifying selection in evolve and resequencing studies. Genome Biol. 20:169. 31416462 10.1186/s13059-019-1770-8 PMC6694636

[jkab324-B96] Wang JB , Lu HL, Leger RJS. 2017. The genetic basis for variation in resistance to infection in the *Drosophila melanogaster* genetic reference panel. PLoS Pathog. 13:e1006260. 28257468 10.1371/journal.ppat.1006260 PMC5352145

[jkab324-B97] Wiberg RAW , Gaggiotti OE, Morrissey MB, Ritchie MG. 2017. Identifying consistent allele frequency differences in studies of stratified populations. Methods Ecol Evol. 8:1899–1909. 29263778 10.1111/2041-210X.12810 PMC5726381

[jkab324-B98] Yang X , Li J, Lee Y, Lussier YA. 2011. GO-Module: functional synthesis and improved interpretation of Gene Ontology patterns. Bioinformatics. 27:1444–1446. 21421553 10.1093/bioinformatics/btr142 PMC3087953

[jkab324-B99] Ye YH , Chenoweth SF, McGraw EA. 2009. Effective but costly, evolved mechanisms of defense against a virulent opportunistic pathogen in *Drosophila melanogaster*. PLoS Pathog. 5:e1000385. 19381251 10.1371/journal.ppat.1000385 PMC2663048

